# Outcomes of ureteroscopy for stone disease in anomalous kidneys: a systematic review

**DOI:** 10.1007/s00345-019-02810-x

**Published:** 2019-05-17

**Authors:** Lisa Lavan, Thomas Herrmann, Christopher Netsch, Benedikt Becker, Bhaskar K. Somani

**Affiliations:** 1 University Hospital Southampton NHS Trust, Southampton, UK; 2 Klinikdirektor Urologie Spital Thurgau AG, Chefarzt Urologie Kantonspital Frauenfeld, Frauenfeld, Thurgau Switzerland; 3 Department of Urology, Asklepios Hospital Barmbek, Rübenkamp 220, 22291 Hamburg, Germany; 4 University of Southampton, Southampton, UK

**Keywords:** Renal anomaly, Horseshoe kidney, Ectopic kidney, Malrotation, Pelvic, Ureteroscopy, Urolithiasis, RIRS

## Abstract

**Introduction:**

Treatment of stone disease in anomalous kidneys can be challenging. As ureteroscopy (URS) has advanced, the number of studies reporting on outcomes of URS for stone disease in anomalous kidneys has increased. Our objective was to perform a systematic review of the literature to evaluate the outcomes of URS for stone disease in this group of patients.

**Methods:**

A Cochrane style review was performed in accordance with the PRISMA guidelines using Medline, EMBASE, CINAHL, Cochrane Library, Scopus and individual urologic journals for all English language articles between inception and June 2018.

**Results:**

Fourteen papers (413 patients) with a mean age of 43 years and a male to female ratio of 285:128 were included. The underlying renal anomaly was horseshoe kidney (*n* = 204), ectopic kidney (*n* = 117), malrotation (*n* = 86), cross fused ectopia (*n* = 2) and others (*n* = 2). With a mean stone size of 16 mm (range 2–35 mm), the majority of stones were in the lower pole (*n* = 143, 34.6%) or renal pelvis (*n* = 128, 31.0%), with 18.9% (*n* = 78) having stones in multiple locations. Treatment modality included the use of flexible ureteroscope in 90% of patients and ureteral access sheath used in 11 studies. With a mean operative time of 61.3 min (range 14–185 min), the initial and final SFR was 76.6% (*n* = 322) and 82.3% (*n* = 340), respectively. The overall complication rate was 17.2% (*n* = 71), of which 14.8% were Clavien I/II and the remaining 2.4% were Clavien ≥ III complications.

**Conclusion:**

Although ureteroscopy in patients with anomalous kidneys can be technically challenging, advancements in endourological techniques have made it a safe and effective procedure. In these patients the stone-free rates are good with a low risk of major complications.

## Introduction

Anomalous kidneys arise from different abnormalities in the embryological development [1]. These may relate to abnormal ascent, fusion, rotation or a combination of these. Whilst the commonest renal anomaly is the horseshoe kidney (HSK) with an incidence of 1 in 400, ectopic kidneys (EK) are reported with an incidence of 1 in 3000, with the incidence of isolated malrotation (MR) less widely reported [1].

These anatomical anomalies not only lead to compromised renal drainage, but also increase the risk of urolithiasis [2–4]. Endourological management is challenging due to these abnormalities leading to difficulties accessing the stone [2]. Treatment such as shockwave lithotripsy (SWL) and percutaneous nephrolithotomy (PCNL) are well described in anomalous kidneys, but can be technically challenging, with success rates often reported to be lower than those in normal kidneys [3–8].

Advances in technology and technique have allowed a broadening of indications for flexible ureterorenoscopy (FURS). The development of smaller calibre ureteroscopes with their increased deflection capability, along with holmium laser fibres and other adjuncts, make FURS an attractive treatment modality for challenging intrarenal anatomy [9].

Recently, the number of studies reporting on the outcomes of ureteroscopy (URS) in anomalous kidneys has increased. However, endoscopic access can be challenging, with complications and stone-free rates (SFR) that are variable across the reported studies. This article aims to review and summarise the efficacy and safety of FURS for urolithiasis in anomalous kidneys.

## Methods

### Search strategy and study selection

**Fig. 1:**
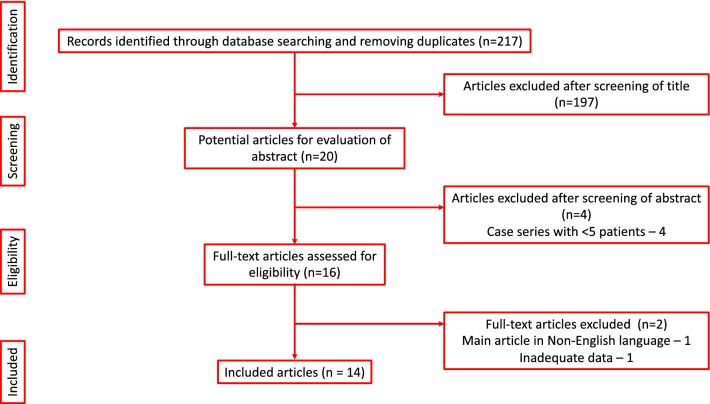
PRISMA flowchart of the included studies

Our systematic review was performed according to Cochrane review guidelines and the preferred reporting items for systematic reviews and meta-analysis (PRISMA) standards [10]. A literature search was conducted using MEDLINE, EMBASE, CINAHL, Scopus, the Cochrane Library and individual urology journals for all English language articles. Search terms used included the following: ‘ureteroscopy’, ‘ureterorenoscopy’, ‘retrograde intrarenal surgery’, ‘RIRS’, ‘URS’, ‘ureteroscopy’, ‘ureteroscopic management’, ‘urolithiasis’, ‘anomalous kidney’, ‘malrotation’, ‘horseshoe kidney’, ‘ectopic kidney’, ‘calculi’ and ‘stone’. The references of identified studies were examined to identify any further potential studies for inclusion. Boolean operators (AND, OR) were used to refine the search. The study period was from inception of databases to June 2018 (Fig. 1).

A cutoff of five patients was set to include studies from centres with minimum relevant endourological experience in managing stones in anomalous kidneys. All original studies were included and where more than one article was available, the study with the longest follow-up was included. Two reviewers (LL and BS) not involved in the original work identified all the studies and those that appeared to fit the inclusion criteria were included for full review. The studies were selected independently, and all discrepancies resolved by mutual consensus.

### Inclusion criteria

1. All English language articles reporting on the outcomes of ureteroscopic management of urolithiasis in anomalous kidneys.
2. Patients of all age groups.

### Exclusion criteria

1. Case reports, review articles and case series with less than five patients.
2. Simulation, animal and laboratory studies.
3. Studies with non-urolithiasis condition or use of treatments other than URS.

### Data extraction and analysis

Of the eligible studies, data were extracted for patient and stone demographics, previous endourological procedures, imaging modality used, operative technique, including laser fibre size and settings used, SFR including stone-free definition, need for further procedures, follow-up protocol and complications, using Clavien–Dindo classification [11]. Data was collated using Microsoft Excel (version 12.2.4). Quality of evidence was assessed, and bias was analysed using the GRADE assessment tool [12].

## Results

After initial identification and screening of 217 articles, 20 abstracts were further evaluated. Of these, on screening of abstracts and full paper, 14 full text articles were included in the final review (Fig. 1). In total, 413 patients with a mean age of 43 years (range 1–78 years) and a male to female ratio of 285:128 were included. The underlying renal anomaly was HSK (*n* = 204), EK (*n* = 117), MR (*n* = 86), cross fused ectopia (*n* = 2) and others (*n* = 4). The majority of these studies (*n* = 13) were retrospective with just one prospective study [26] (Table 1).

**Table 1:** Patient demographics and case mix of the included studies

References	Study design	Total patients	HSK	EK	MR	Other	Mean age, years (range)	Male	Female
Weizer et al. [13]	Retrospective	8	4	4	0	0	50.6 (35–69)	6	2
Molimard et al. [14]	Retrospective	17	17	0	0	0	34.7 (16–52)	14	3
Atis et al. [15]	Retrospective	20	20	0	0	0	40.9 (NR)	12	8
Bozkurt et al. [16]	Retrospective	26	0	26	0	0	41.1 (7–72)	19	7
Oḡuz et al. [17]	Retrospective	24	0	0	24	0	39.8 (1–71)	18	6
Urgulu et al. [18]	Retrospective	25	3	11	4	5	39.4 (NR)	17	8
Ding et al. [19]	Retrospective	16	16	0	0	0	42.9 (22–66)	13	3
Blackburn et al. [20]	Retrospective	20	20	0	0	0	48.1 (29–78)	13	7
Gokce et al. [21]	Retrospective	23	23	0	00	0	42.5 (16–78)	18	5
Bansal et al. [22]	Retrospective	9	9	0	0	0	NR	7	2
Ergin et al. [23]	Retrospective	101	36	33	32	0	39.0 (1–72)	68	33
Singh et al. [24]	Retrospective	25	5	14	5	1	38.28 (NR)	17	8
Legemate et al. [25]	Retrospective	86	43	27	16	0	49.2 (NR)	57	29
Astolfi et al. [26]	Prospective	13	8	0	5	0	46.1 (NR)	6	7
Total		413	204	117	86	6	43.4	285	128

*HSK* horseshoe kidney, *EK* ectopic kidney, *MR* malrotation

The mean stone size reported from 12 studies was 16 mm (range 2–35 mm). In the majority of patients the stone location was in the lower pole (*n* = 143, 34.6%) or renal pelvis (*n* = 128, 31%), with 18.9% (*n* = 78) having stones in multiple locations (Table 2). Pre-operative imaging included a combination of modalities [intravenous urogram (IVU), plain abdominal KUB XR (AXR), ultrasound scan (USS) or CT scan], although two studies used non-contrast computerised tomography (NCCT) as the only imaging modality [14, 24] (Table 3). A total of 126 (30.5%) patients had a history of previous endourological intervention [13–19, 22, 25] (Table 3).

**Table 2:** Stone size and location in the included studies

References	Stone size mean (mm)/[mm^2^]	Stone size range (mm)/[mm^2^]	Single stone	Multiple stones	Stone position, lower pole	Stone position, midpole	Stone position, upper pole	Stone position, renal pelvis	Stone position, mixed	Stone position, upper ureter
Weizer et al. [13]	14	3–20	5	3	4	0	2	5	(3)	0
Molimard et al. [14]	16	7–35	7	10	–	–	–	7	10	0
Atis et al. [15]	17.8	± 4.5	5	15	9	7	4	5	(5)	0
Bozkurt et al. [16]	17	10–28	21	5	7	0	0	14	5	0
Oḡuz et al. [17]	13.5	5–30	24	0	9	3	2	10	0	0
Urgulu et al. [18]	[194.7]	[85–393]	19	6	14	4	7	7	(6)	0
Ding et al. [19]	29.8	17–42	4	12	1	1	0	2	12	0
Blackburn et al. [20]	8.4	2–25	17	3	10	NR	NR	NR	NR	0
Gokce et al. [21]	17.1	6–25	14	9	6	0	0	17	0	0
Bansal et al. [22]	15.4	NR	6	3	12	0	0	0	0	0
Ergin et al. [23]	16.1	NR	NR	NR	35	16	14	45	(9)	0
Singh et al. [24]	14.7	± 4.1 mm	15	10	11	8	5	11	(12)	2
Legemate et al. [25]	[84]	[4–117]	70	16	18	3	2	3	15	13
Astolfi et al. [26]	12.2	6–22	12	1	7	2	0	2	1	0
Total [mean]	[16.0]		219	93	143	44	36	128	78	15

**Table 3:** Data on pre-operative variables

References	Pre-operative imaging	Pre-operative urine MC and S	Peri-operative antibiotics	Pre-operative stent	Previous SWL	Previous PCNL	Previous open procedure	Previous URS	> 1 previous procedure
Weizer et al. [13]	IVU or NCCT	NR	NR	1 12.5%)	6	0	1	0	1
Molimard et al. [14]	NCCT	Yes	NR	4 (23.5%)	8	4	2	3	NR
Atis et al. [15]	AXR and IVU or US	Yes	Yes	0	4	4	4	0	4
Bozkurt et al. [16]	NCCT or IVU	Yes	NR	NR	9	0	1	0	0
Oḡuz et al. [17]	AXR, IVU, US or NCCT	Yes	Yes	NR	12	0	0	4	0
Urgulu et al. [18]	IVU and CT	Yes	NR	NR	7	2	2	1	NR
Ding et al. [19]	AXR and IVU or NCCT	NR	Yes	NR	7	1	0	0	1
Blackburn et al. [20]	NR	NR	NR	NR	NR	NR	NR	NR	NR
Gokce et al. [21]	AXR and USS or NNCT	Yes	Yes	NR	NR	NR	NR	NR	NR
Bansal et al. [22]	AXR, IVU, USS or NCCT	NR	Yes	NR	2	5	0	0	0
Ergin et al. [23]	IVU, USS or NCCT	Yes	NR	NR	NR	NR	NR	NR	NR
Singh et al. [24]	CTU	Yes	NR	5 (20%)	NR	NR	NR	NR	NR
Legemate et al. [25]	AXR, IVU or NCCT	Yes	Yes	18 (24.7%)	20	12	0	15	NR
Astolfi et al. [26]	AXR or NCCT	NR	NR	11 (84.6%)	NR	NR	NR	NR	NR
Total				40 (29.4%)	75 (59.2%)	28 (22.2%)	10 (7.9%)	23 (18.3%)	

*AXR* plain abdominal X-ray, *IVU* intravenous urogram, *USS* ultrasound scan, *NCCT* non-contrast computerised tomography, *CTU* computerised tomography urogram, *NR* not reported, *SWL* shockwave lithotripsy, *PCNL* percutaneous nephrolithotomy, *URS* ureteroscopy

While semirigid URS was used in 41 (10%) cases, FURS was used in 90% of cases. Of the reported studies, a pre-operative stent was reported in 26.4% (range 12.4–84.6%, 40/136 patients) [13–15, 24–26]. Placement of ureteral access sheath (UAS) was reported in 11 studies [13–19, 21, 22, 24, 25]. The success rates for UAS placement varied from 50 to 100% across studies (Table 4). Fragmentation device was reported in 13 studies, of which 12 used holmium laser lithotripsy for all of their patients. A range of fibre sizes and energy settings was reported. The mean operative time was 61.3 min (range 14–185 min). While six studies report a post-operative stent placement in all of their patients [13, 14, 19, 21, 22, 26], in the remaining studies this varied from 46.2 to 84% and was at the surgeon’s discretion [15–17, 23–25]. The mean hospital stay across studies was 1.7 days (range 0.5–9 days) (Table 4).

**Table 4:** Intraoperative and post-operative data from included studies

References	Anaesthesia	Procedure	Holmium laser	Fibre size (μm)	Energy setting (J)	Energy setting (Hz)	Access sheath used	Post-operative stent	Mean operative time, min (range)	Length of stay, days (range)
Weizer et al. [13]	NR	FURS	Yes	200	0.6–1.0	6–10	4 (50%)	8 (100%)	126 (90–185)	NR
Molimard et al. [14]	GA	FURS	Yes	150 or 365	0.8–1.2	8–12	17 (100%)	17 (100%)	92 (45–140)	1.7 (1–3)
Atis et al. [15]	GA	SRU and FURS	Yes	273	0.6–1.0	5–10	20 (100%)	15 (75%)	40.5	1.4
Bozkurt et al. [16]	GA	FURS	Yes	200	0.8	10	0	12 (46%.	52.1 (30–120)	2.7 (1–9)
Oḡuz et al. [17]	GA	SRU and FURS	Yes	273	NR	NR	20 (83%)	17 (71%)	48.7 (18–135)	1.5 (1–5)
Urgulu et al. [18]	GA	FURS	Yes	200 or 273	NR	NR	18 (72%)	NR	48 (14–115)	NR
Ding et al. [19]	Spinal	SRU and FURS	Yes	200	0.8–1.2	10–15	16 (100%)	16 (100%)	92 (14–127)	0.8 (0–3)
Blackburn et al. [20]	NR	NR	NR	NR	NR	NR	NR	NR	NR	NR
Gokce et al. [21]	GA	FURS	Yes	NR	0.8–1.2	8–12	23 (100%)	23 (100%)	NR	1.8 (1–3)
Bansal et al. [22]	GA	FURS	Yes	200	0.6–0.8	10–15	9 (100%)	9 (100%)	84.2	NR
Ergin et al. [23]	GA	SRU and FURS	Yes	170 or 200	NR	NR	NR	50 (50%)	47.1	1.9
Singh et al. [24]	GA	FURS	Yes	200 or 365	0.5–1.0	10–15	25 (100%)	21 (84%)	74	2.48
Legemate et al. [25]	NR	SRU alone 47.7% FURS 32.6% Combination 17.4%	57%	NR	NR	NR	29 (71%)	71 (84%)	58 (30–120)	1 (0.5–5)
Astolfi et al. [26]	GA	SRU and FURS	Yes	200 or 273	NR	NR	NR	13 (100%)	NR	NR

*GA* general anaesthetic, *SRU* semirigid ureteroscopy, *FURS* flexible ureterorenoscopy, *NR* not reported

With no universal definition of SFR between studies, their follow-up imaging also varied and sometimes even within each study. While ten studies did the post-operative imaging after 4 weeks [13–19, 21, 22, 24], one study did it after 3 months [26] and the remaining three did not mention the time interval for follow-up imaging [20, 23, 25] (Table 5).

**Table 5:** Post-operative outcomes from included studies

References	Definition of success	Post-op imaging modality	Imaging time interval (weeks)	Overall success rate (%)	Success after single procedure	Auxiliary procedures required	Readmission	Complications Clavien I–II	Complications Clavien ≥ III
Weizer et al. [13]	Stone free	AXR and IVU or NCCT	4–12	75.0	75.0	0	NR	0	0
Molimard et al. [14]	RF ≤ 3 mm	AXR and USS or NCCT	4 - 6	88.2	53.0	7 URS	1	8	0
Atis et al. [15]	RF < 4 mm	IVU and USS (NCCT if RF)	4	80.0	70.0	6 SWL	0	5	0
Bozkurt et al. [16]	RF ≤ 2 mm	NCCT	4	84.7	NR	NR	NR	3	2
Oḡuz et al. [17]	RF ≤ 3 mm	IVU and USS (NCCT if RF)	4	83.3	75.0	1 SWL 1 URS	1	11	2
Urgulu et al. [18]	Complete clearance	NCCT	4	88.0	64.0	6 URS 3 SWL	NR	3	0
Ding et al. [19]	Not defined	AXR and USS	4	87.5	62.5	6 URS	NR	3	0
Blackburn et al. [20]	RF < 4 mm	AXR or CT	NR	84.0	NR	NR	NR	NR	NR
Gokce et al. [21]	RF < 3 mm	AXR and/or USS/NCCT	2 - 6	73.9	NR	NR	0	4	0
Bansal et al. [22]	RF ≤ 4 mm	AXR and USS or NCCT	4	88.9	67.7	3 URS	1	4	0
Ergin et al. [23]	RF < 3 mm	NR	NR	76.9	NR	8 URS	NR	12	2
Singh et al. [24]	RF < 2 mm	AXR and USS	4	88.0	72.0	3 PCNL	NR	5	1
Legemate et al. [25]	RF ≤ 1 mm	AXR and USS or NCCT	NR	58.3	NR	15	12	2	3
Astolfi et al. [26]	RF < 2 mm	AXR or NCCT	12	75.0	NR	NR	0	1	0
Overall				82.3% (*n* = 340)	76.6% (*n* = 322)	18	15	61 (14.8%)	10 (2.4%)

*RF* residual fragments, *AXR* plain abdominal X-ray, *IVU* intravenous urogram, *USS* ultrasound scan, *NCCT* non-contrast computed tomogram, *URS* ureteroscopy, *SWL* shockwave lithotripsy, *PCNL* percutaneous nephrolithotomy

The initial and final SFR was 76.6% (*n* = 322) and 82.3% (*n* = 340), respectively, with 18 patients needing ancillary treatment which was a mix of repeat URS or SWL or PCNL (Table 5). Three studies reported the demographics and outcomes of HSK, EK and MR individually [18, 23, 25] (Table 6).

**Table 6:** Studies reporting on outcomes for individual data for HSK, EK and MR

		Mean stone burden [range]	Percentage of lower pole stones (%)	Success after single procedure	Overall success (%)
Urgulu et al. [18]	HSK (*n* = 3)	253 mm^2^ ± 103.7	50.0	66.7%	66.7
	EK (*n* = 13)	237.7 mm^2^ ± 94.4 (lumbar) 168.8 mm^2^ ± 101.7 (pelvic)	57.1 33.3	61.5%	100
	MR (*n* = 4)	201.3 mm^2^ ± 109.5	75.0	100%	100
Ergin et al. [23]	HSK (*n* = 36)	17.8 mm ± 4.5	30.6	NR	72.2
	EK (*n* = 33)	17.0 mm ± 5.1	36.4	NR	83.6
	MR (*n* = 32)	13.4 mm ± 3.7	37.5	NR	75.0
Legemate et al. [25]	HSK (*n* = 23)	70 mm^2^ [46–134]	52.1	NR	77.3
	EK (*n* = 10)	120 mm^2^ [79–263]	30.0	NR	20.0
	MR (*n* = 8)	62 mm^2^ [0–148]	37.5	NR	71.4

*HSK* horseshoe kidney, *EK* ectopic kidney, *MR* malrotation

Overall, 71 (17.2%) complications were reported of which 61 (14.8%) were Clavien–Dindo ≤ II, and 10 (2.4%) were Clavien–Dindo ≥ III. The Clavien I/II complications included stent symptoms (*n* = 7), haematuria (*n* = 15), post-operative pyrexia (*n* = 21) and confirmed urinary tract infection (*n* = 6). Of the nine Clavien III complications, surgical intervention for ureteric colic accounted for seven of these and the remaining two interventions were not specified. The single Clavien IV complication occurred in one of the largest studies [25], where an obese patient with a large stone (262 mm [2]) and prolonged operating time (121 min) developed sepsis with acute renal failure. This patient was treated with percutaneous nephrostomy, antibiotics and intensive care support (ICU) (Tables 5, 7).

**Table 7:** Complications graded as per Clavien–Dindo classification

References	Clavien I–II	Clavien ≥ III
Weizer et al. [13]	None	None
Molimard et al. [14]	Stent symptoms *n* = 6 Haematuria *n* = 1 Pyelonephritis *n* = 1	None
Atis et al. [15]	Post-operative pyrexia *n* = 3 Haematuria *n* = 2	None
Bozkurt et al. [16]	Post-operative pyrexia *n* = 1 Haematuria *n* = 1 Urinary tract infection *n* = 1	Ureteric colic requiring JJ stent *n* = 2
Oḡuz et al. [17]	Post-operative pyrexia *n* = 2 Ureteric colic (conservative management) *n* = 9	Ureteric colic requiring surgical intervention *n* = 2
Urgulu et al. [18]	Urosepsis *n* = 1 Pyelonephritis *n* = 1 Ureteric colic (conservative management) *n* = 1	None
Ding et al. [19]	Post-operative pyrexia *n* = 3	None
Blackburn et al. [20]	Complications not reported	Complications not reported
Gokce et al. [21]	Haematuria *n* = 3 Post-operative pyrexia *n* = 1	None
Bansal et al. [22]	Post-operative pyrexia *n* = 2 Stent symptoms *n* = 1 Pyelonephritis *n* = 1	None
Ergin et al. [23]	Haematuria *n* = 7 Post-operative pyrexia *n* = 5	Ureteric colic requiring JJ stent *n* = 2
Singh et al. [24]	Post-operative pyrexia *n* = 3 Urinary tract infection *n* = 2	Ureteric colic requiring JJ stent *n* = 1
Legemate et al. [25]	Post-operative pyrexia *n* = 1 Urosepsis *n* = 1	IIIa not defined *n* = 1 IIIb not defined *n* = 1 IVa Urosepsis requiring nephrostomy and ITU support *n* = 1
Astolfi et al. [26]	Haematuria *n* = 1	None

### Quality assessment of the included studies

**Fig. 2:**
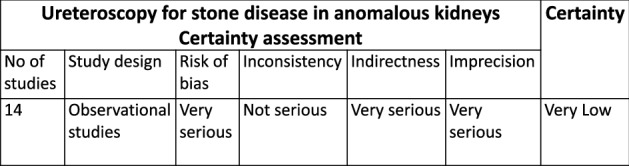
Risk of bias analysis

Of the 14 studies included, there was only one prospective study [26], with all others based on retrospective observational case series. The overall quality of evidence was graded as ‘very low’ and risk of bias ‘very high’ as detailed in Fig. 2.

## Discussion

### Meaning of the study

The incidence of anomalous kidneys is relatively low with mostly small retrospective studies reporting on the outcomes of surgery for urolithiasis in these patients. However, in experienced hands ureteroscopy can offer good SFR with a low risk of major (Clavien ≥ III) complications even for large stones. It seems that over the last decade, there have been more studies reporting on the outcomes of FURS in this setting.

### Comparison of studies reporting on FURS for renal anomalies

Weizer et al. [13] reported the first series on ureteroscopic management of renal calculi in eight patients with anomalous kidneys (four HSK, four EK) with stones up to 2 cm. They report the use of UAS to straighten the tortuous ureter, relocation of stone to a more favourable location and extraction of fragments leading to an overall success rate of 75% with none of the patient requiring auxiliary treatment.

Molimard et al. [14] reviewed the outcomes of FURS and holmium lasertripsy in 17 patients with horseshoe kidneys. They used UAS in all patients with automatic flow irrigation at 100 cm H_2_O to improve visualisation. While the laser settings varied upon clinical situations, stone repositioning and extraction was used for clearance. They also advised patients on force fluid intake post-operatively to facilitate passage of small fragments. However, staged FURS was needed in larger stones and those in difficult locations, with an overall success rate of 88.2%.

Atis et al. [15] described FURS in 20 patients with horseshoe kidneys. They performed a semirigid URS in all cases to initially dilate the ureter before placement of a UAS. They recommend stone relocation where possible and to use dusting setting (high frequency, low energy) for stone treatment. Failure was significantly higher in the lower pole and larger stones.

Bozkurt et al. [16] investigated the outcomes in 26 patients with pelvic ectopic kidneys. Stone relocation and dusting method of stone treatment was used; however, a UAS was not used due to short tortuous ureter. Although the treatment was successful in 84.7%, it failed in patients with unfavourable infundibulopelvic anatomy.

Oḡuz et al. [17] used FURS for treating kidney stones in 24 patients with isolated anomaly of kidney rotation, excluding HSK and EK. They used semirigid URS to passively dilate the ureter and placed a UAS in 83% of patients, with an initial and final SFR of 75% and 83.3%, respectively.

Urgulu et al. [18] used FURS for stone disease in 25 patients with anomalous kidneys, including 1 patient with cross fused ectopia. They suggest the use of paediatric 9.5–11.5 F UAS in pelvic kidneys to overcome the difficulties of short tortuous ureters. The size of laser fibre and energy settings were determined intra-operatively according to stone size, location and composition.

Ding et al. [19] reviewed the efficacy of FURS in 16 patients with HSK. Semirigid URS and UAS were used in all patients. Stone relocation was seen to increase the SFR as well as protecting the ureteroscope by minimising the duration of scope deflection. With six patients needing a repeat FURS, the initial and final SFR was 62.5% and 87.5%, respectively.

Gokce et al. [20] compared the outcomes of SWL and FURS for treatment of stone disease in 67 patients with HSK, with similar patient and stone demographics between the groups. They recommend placing the patients in a slight Trendelenburg position to encourage stones to fall into upper calyces. They also used UAS, repositioned lower pole stones, used automatic flow irrigation at 100 cm H_2_O to improve visualisation and placed a ureteric stent as well as a urethral catheter in all patients to maximise drainage post-operatively. The SFR rate was significantly higher (*p* = 0.039) in the FURS group (73.9%) compared to the SWL group (47.7%) with no significant difference in complication rates between the groups.

Bansal et al. [22] treated nine patients (12 renal units) with HSK and lower calyceal stones using FURS. They used UAS for all patients to optimise vision, keep a low intrarenal pressure and extract fragments. In cases where UAS placement was not possible, patients were stented and booked for a second planned procedure. With a stone dusting laser setting, the initial and final SFR was 67.7% and 88.7%, respectively.

Ergin et al. [23] reported on 101 patients who underwent surgery for urolithiasis in anomalous kidneys over a 10-year period. Surgical techniques included FURS for stones less than 2 cm and PCNL for stones greater than 2 cm, or laparoscopic pyelolithotomy for large stones in ectopic kidneys. The overall SFR for HSK in the FURS group was 72.2% compared to 90% in the PCNL group; however, 14 patients in the PCNL group required a second procedure. In the EK group, FURS was compared to laparoscopic pyelolithotomy, although all stones in the laparoscopic pyelolithotomy group were in the renal pelvis (*n* = 9). The SFR rate for EK was 83.6% and 100% for FURS and laparoscopic pyelolithotomy, respectively. Finally, SFR for isolated rotational anomalies for FURS and PCNL was 75% and 83.3%, respectively. The overall SFR for FURS in all renal anomalies combined was 76.9%.

Singh et al. [24] presented outcomes of FURS in 25 patients with various renal anomalies and stones < 2 cm. UAS and stone relocation to a favourable position was used in all patients. Laser settings were adjusted with dusting setting preferred for stone treatment. Patients were given an alpha blocker post-operatively and encouraged to increase their fluid intake to improve stone passage.

Legemate et al. [25] reviewed data from the Clinical Research Office of the Endourological Society (CROES) URS Global Study and, of the 11,885 patients included, 86 patients were identified with anomalous kidneys that underwent URS for both renal and ureteric stones. The SFR for patients with and without pre-operative stent was 67% and 78%, respectively, and with and without UAS was 66% and 50%, respectively. Although the mean stone burden was highest in the EK group (120 mm^2^), the SFR for HSK, MR and EK groups was 77%, 71% and 20%, respectively. The SFR decreased in all three groups for patients in case the stone burdens were greater than 80 mm^2^.

Astolfi et al. [26] collected prospective data on patients with anomalous kidneys undergoing FURS over a 6-year period and reported outcomes for 13 patients with an SFR of 75%. Semirigid ureteroscopy was performed initially and UAS use was preferred. Laser settings were adjusted according to stone location and composition with nitinol baskets used to relocate stones from unfavourable positions and to remove fragments.

The anatomical variations of anomalous kidneys can lead to difficulties in either localising or accessing stones for treatment and therefore a higher complication rate may be expected compared to surgery for stones in normally formed kidneys [27]. Bas et al. [28] retrospectively analysed data on 1395 patients undergoing FURS for renal or proximal ureteric calculi and attempted to determine predictive factors affecting complication rates. On multivariate analysis, the only significant predictive factor was the presence of congenital renal abnormalities.

The overall complication rate from the included studies was 17.2%. Out of these complications, only 2.4% were Clavien > III complications most of which related to re-intervention for ureteric colic. There was one Clavien IVa complication where a patient with urosepsis and acute renal failure received a nephrostomy and was transferred to the intensive care unit (ICU).

### Tips and practical stepwise guidance for management from the included studies

**Fig. 3:**
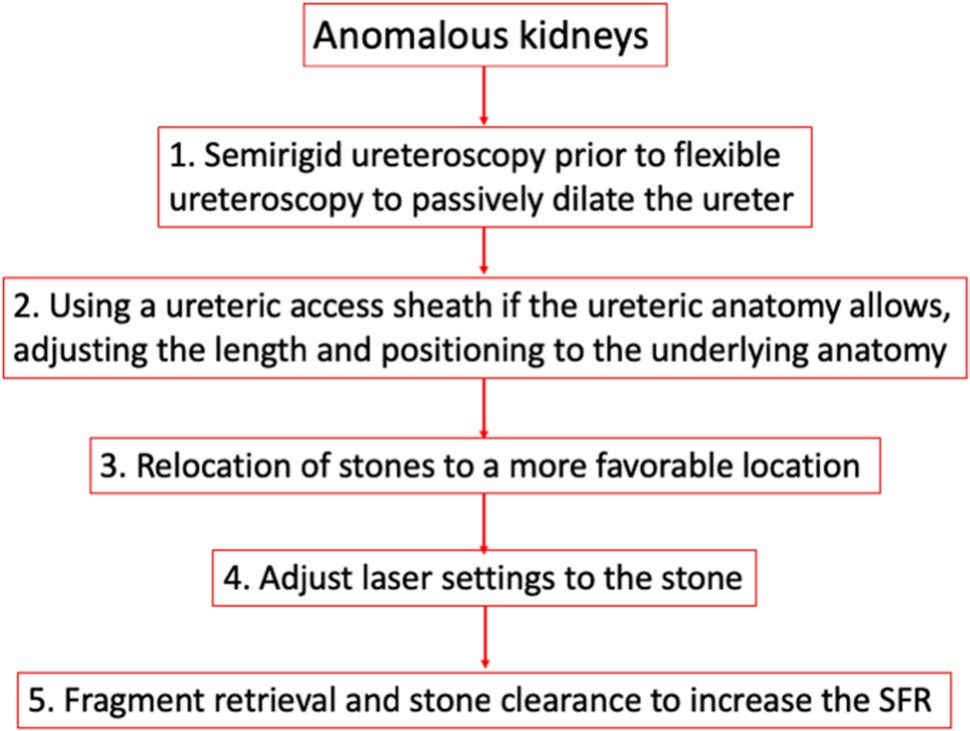
Tips and practical guidance for management

Based on the included studies, there were certain tips and recommendations for ureteroscopy for stone disease in anomalous kidneys (Fig. 3). In a stepwise manner this included:

1. Performing a semirigid URS prior to FURS to passively dilate the ureter.
2. Using a UAS if the ureteric anatomy allowed it and choosing a smaller length in pelvic kidneys. In EK, it should be adjusted to mid to lower ureter, or in a position such that the scope can flex in the pelvicalyceal system.
3. Relocation of stones from an unfavourable to a more favourable position.
4. Adjusting laser setting according to stone composition, but dusting seemed to be the preferred mode of stone treatment.
5. Fragment retrieval and stone clearance to increase the SFR.

### Comparison of URS with PCNL and SWL in management of stones in anomalous kidneys

The anatomical variation of anomalous kidneys presents technical challenges to access stones for treatment irrespective of the surgical technique undertaken [29].

Whilst SWL has the advantage of being non-invasive and avoids the need for general anaesthesia, stone localisation can be difficult due to the overlying bony structures or due to interposed bowel gas. The skin to stone distance is often increased and, even if SWL was successful in fragmenting the stone, impaired drainage can hinder the passage of the fragments, resulting in reduced SFR [27].

Ray et al. [6] reviewed the data of 41 patients with HSK undergoing SWL for renal stones. The success rate defined as being stone free or asymptomatic with residual fragments < 4 mm after single treatment was only 25%, increasing to 63.6% with additional treatments. They observed very little clinical benefit of offering more than two SWL sessions and multivariate analysis found stone burden, stone position and patient body mass index to be prognostic for SWL success. Sheir et al. [7] reported on their experience of SWL in 198 patients who were treated for a mean stone size of 13.54 mm (± 5.49). The overall SFR was 72.2% with 3.2% of patients who developed a steinstrasse. Tunc et al. [8] assessed the outcomes of 150 patients with anomalous kidneys and reported an overall SFR of 68% at 3 months. Stone size drove the success rate with SFR of 34% and 92% for stones > 30 mm and < 10 mm in size, respectively.

PCNL offered higher stone clearance rates compared to SWL, but with a higher risk of associated complications. Due to the anatomical variations and abnormal relationship to the adjacent organs (especially bowel), there was an increased risk of iatrogenic injury during percutaneous access in PCNL, and access tracts were often longer. Abnormal vasculature was also common that must be considered in pre-operative planning [27]. Symons et al. [4] reviewed the 15-year outcomes of all patients who underwent surgical treatment for HSK. Of the 55 patients identified, the majority (85.5%) underwent PCNL, with an SFR of 77% after a single procedure. Tepeler et al. [3] analysed factors affecting outcomes of PCNL in 53 patients with HSK. For a mean stone size of 28.4 mm, the initial and final SFR was 66.7% and 90.7%, respectively. While auxiliary treatments increased SFR, the only factor affecting success rates on multivariate analysis was stone multiplicity.

### Strengths, limitations and areas of future research

This systematic review comprehensively summarises the evidence for the role of URS in the setting of anomalous kidneys. Apart from the outcomes, it looks at tips and practical stepwise guidance provided from the included studies. Furthermore, the results can potentially set a benchmark for patient counselling and future research. The quality of included studies was poor with a high risk of bias and based mostly on small retrospective series; however, given the rarity of this condition, our review provides valuable insight, helps to condense the literature and might offer pitfalls and treatment strategies to endourologists. Although the reported complications in anomalous kidneys were higher, the rates of major complications were not different compared to URS in anatomically normal kidneys [30]. Future studies should also look at the cost comparison of the different treatment modalities [31–33]. A lack of standardised methods of data collection and reporting made it difficult to compare or combine the outcomes [34]. Retrograde intrarenal surgery is now being done for complex patients including those with morbid obesity, pregnancy and paediatric patients [35–37]. Improved training, flexible ureteroscopy technology and advances in laser have led to this procedure being successful in those with anomalous kidneys [38–40].

Patients with stone disease in anomalous kidneys need individualised management and probably should involve an interdisciplinary treatment with interventional radiology colleagues with interventions carried out in high volume endourology centres. Although randomised trials between treatment modalities would be difficult given the rarity of this condition, perhaps large prospective multi-centric studies with long-term follow-up and standardised references would be able to provide with high-quality insightful data.

## Conclusion

Although URS in patients with anomalous kidneys can be technically challenging, advancements in endourological techniques have made it a safe and effective procedure. In these patients, the stone-free rates are good with a low risk of major complications.
